# Hajdu-Cheney Syndrome: Report of a Case in Spain

**DOI:** 10.3390/diagnostics12030566

**Published:** 2022-02-23

**Authors:** Jonathan Cortés-Martín, Juan Carlos Sánchez-García, Beatriz Piqueras-Sola, Raquel Rodríguez-Blanque, María Isabel Tovar-Gálvez, Lourdes Díaz-Rodríguez

**Affiliations:** 1 Andalusia Research Plan, Junta de Andalucía, Research Group CTS1068, School of Nursing, Faculty of Health Sciences, University of Granada, 18071 Granada, Spain; jcortesmartin@ugr.es (J.C.-M.); jsangar@ugr.es (J.C.S.-G.); bpiquerassola@gmail.com (B.P.-S.); matoga@ugr.es (M.I.T.-G.); cldiaz@ugr.es (L.D.-R.); 2 School of Nursing, Faculty of Health Sciences, University of Granada, 18071 Granada, Spain; 3 Hospital University Virgen de las Nieves, 18014 Granada, Spain; 4 School of Nursing, Faculty of Health Sciences, University of Granada, Ceuta Campus, 51001 Ceuta, Spain

**Keywords:** Hajdu-Cheney syndrome, rare diseases, acroosteolysis, osteoporosis, bone re-sorption

## Abstract

This paper describes the case of a 54-year-old woman diagnosed with Hajdu–Cheney syndrome, who presents with characteristic craniofacial dysmorphia, short stature, premature loss of teeth, developmental skeletal disorders, fibrocystic mastopathy, bilateral hearing loss and an intermittent mild neutropenia. The patient received treatment with bisphosphonates and was awaiting evaluation for surgical arthroplasty of both hips when she suffered a motor vehicle accident, which led to a rapid progression in her disease by increasing her degree of dependence for most activities of daily living. The clinical presentation and radiologic findings seen in this case confirm the three main features of the syndrome: phenotypic variability, an age-dependent progression and the presence of generalized osteoporosis and acroosteolysis of distal phalanges. The main objective of the manuscript is to describe a new case of a patient diagnosed with Hajdu–Cheney syndrome. Due to the low prevalence of the syndrome and the small number of cases reported in the scientific literature, obtaining a complete description and a global perspective of the disease is complex.

## 1. Introduction

Hajdu-Cheney syndrome (HCS) is a rare genetic disease. It is registered in the database of the OMIM project with reference number 102500 and in ORPHANET under the reference ORPHA955. This disease mainly affects the connective tissue and belongs to the osteolysis syndromes group [1]. It is also known as acro-dento-osteo-dysplasia, acroosteolysis with osteoporosis and changes in the skull and mandible, arthro-dento-osteo-dysplasia and serpentine fibula–polycystic kidney syndrome. It is caused by a heterozygotic mutation of the gene NOTCH2 [2] located on chromosome 1p13-p11 and follows an autosomal-dominant inheritance pattern [3], although descriptions of cases with sporadic mutations can be found [4]. The prevalence of this disease is less than one person in one million (<1/1,000,000).

N. Hajdu first described the disease in 1948 [5], and the description was completed at a later date by D. Cheney in 1965 [6]. Since then, approximately 100 cases have been reported in the scientific literature allowing for the identification of a series of general features, which are shared by all patients, such as a phenotypic variability, an age-dependent progression and the presence of generalized osteoporosis and acroosteolysis of distal phalanges as well as other clinical manifestations [7].

The phenotypic variability [8] is the consequence of the variability of the expression of NOTCH2; hence, patients diagnosed with this disease may present with clinical differences between them. Moreover, this disease presents a wide and specific clinical spectrum so it may be difficult to encounter in full in a single patient.

This disease has a degenerative nature [9]; therefore, the clinical manifestations worsen over time with the onset of many changes from early childhood to late adulthood.

The osteolysis of distal phalanges and generalized osteoporosis [10] found in all cases with HCS is accompanied by a series of clinical manifestations that, as we previously stated, may vary between patients.

These differences include cranial alterations [5] such as dolichocephaly, delayed suture closure, presence of multiple wormian bones, absent frontal sinuses, thickened dome of the skull, occipital prominence, bathrocephaly, elongated sella turcica and micrognathia, which may lead to complications such as basilar invagination [11]), hydrocephalus [12] and syringomyelia [13]; facial alterations such as hypertelorism, sinofridia, thick hair, low-set ears, elongated philtrum, small jaw, high-arched palate, premature denture loss [14], unusually deep voice and hirsutism; and musculoskeletal alterations such as kyphoscoliosis, short stature, fractures of long bones, acroosteolysis [15], progressive distal bone resorption, joint laxity, bone demineralization, and osteoporosis [16]. Other clinical manifestations may include delayed motor development, hearing loss, changes of the voice, congenital heart disease [17], alterations of the respiratory, renal and digestive systems, plantar ulcers and hernias. It must be highlighted that there is a subgroup of patients who present with serpentine fibula and polycystic kidneys [18,19].

The definitive diagnosis is reached by genetic sequencing [20], although the initial diagnosis is established based on the observation of external appearance and the radiological findings [21]. There are certain overlapping features with other diseases such as scleroderma, sarcoidosis, progeria, pycnodysostosis, Whyte-Hemingway, Winchester and Alagille syndrome, which, on occasions, may have to be included in the differential diagnosis.

Currently, there is no definitive or effective pharmacological treatment for HCS, although there are projects underway studying this aspect [22]. At present, the treatment for this disease is centered around the management of complications and underlying problems with the aim of improving the patient’s quality of life and life expectancy.

There are currently more than 7000 rare diseases in the world, of which only 800 have minimal scientific knowledge. In general, rare diseases and in particular, Hajdu–Cheney syndrome, have a low prevalence and few registered cases. The study population sample is dispersed, with a variable phenotype, little-described clinical symptoms and different evolution.

Considering the basic characteristics of this disease, a field of study with many aspects to be explored, can be seen, highlighting aspects such as the complete definition of the phenotype and clinical symptoms through the study of existing cases.

## 2. Patient Information

**Figure 1:**
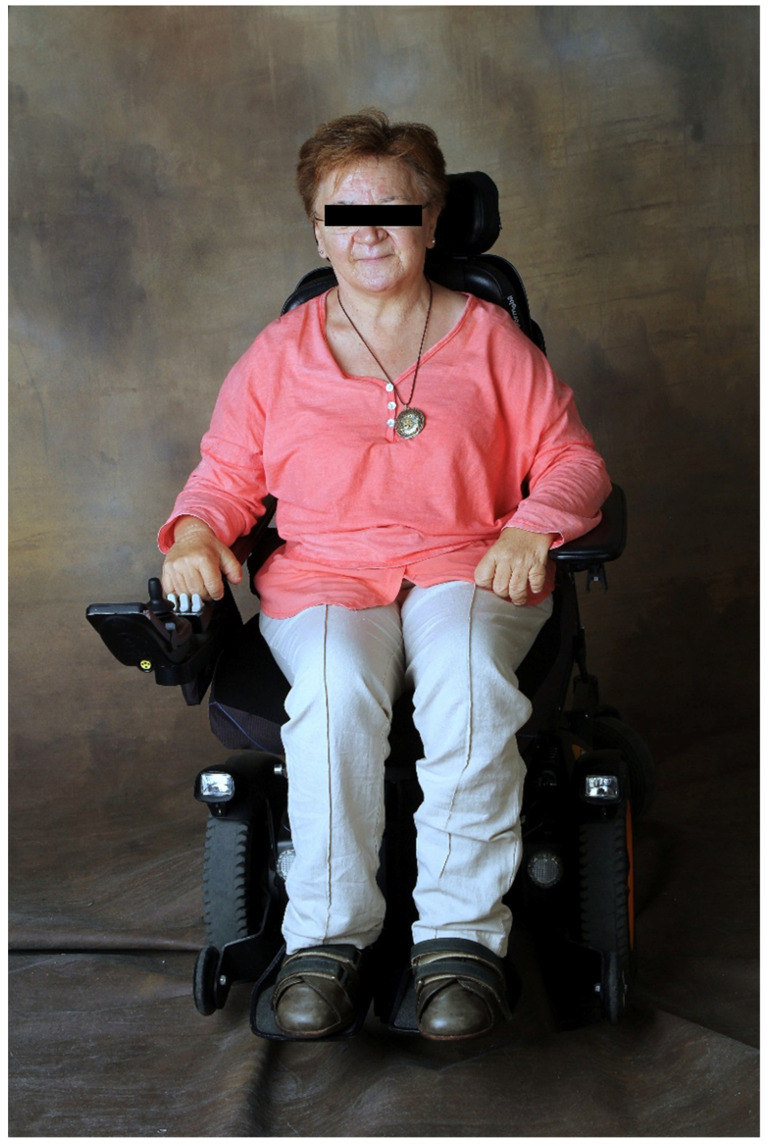
Current photograph of the patient.

The patient was a 54-year-old Caucasian woman (Figure 1). Her mother had no complications during pregnancy and none were present at birth. There was no family history of similar clinical manifestations and she was the youngest of four sisters. She had no children of her own.

The patient was diagnosed with congenital acroosteolysis or Hajdu–Cheney syndrome, osteoporosis, disabling coxarthrosis with acetabular protrusion, musculoskeletal alterations, flat feet, short stature, dysplastic facies, dorsal hyperkyphosis, vitamin D deficiency, low bone density in the trabecular bone, fibrocystic mastopathy, bilateral hearing loss, intermittent mild neutropenia, dysphonia with mild sleep apnea without oximetric repercussions, chronic gastritis and constipation, premature loss of teeth, and adjustment disorder with a longstanding depressive disorder.

The patient was born in 1967. The first clinical registry was obtained at the age of six years after a visit to the Nuestra Señora del Prado Hospital in Talavera de la Reina (Toledo, Spain). After clinical examination, the patient was found to have a moderate psychomotor delay, dysplastic facies, alterations of the extremities and delay in stature closure. The first diagnostic suspicion was pycnodysostosis.

**Figure 2:**
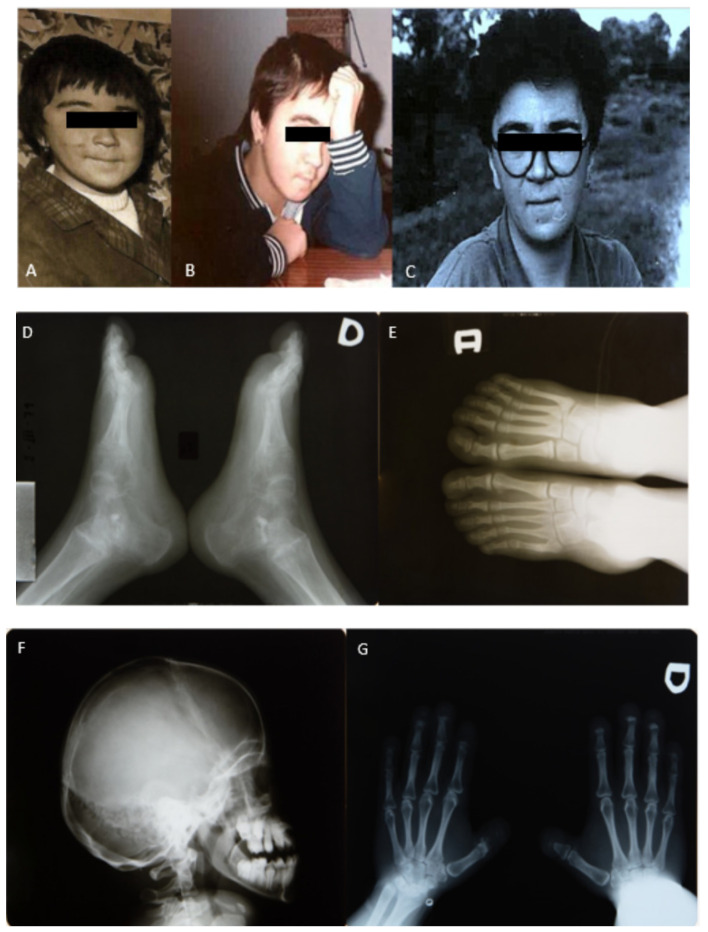
Childhood photographs. Radiologic controls 1977–79. (**A**–**C**) Portraits of childhood. (**D**) Lateral anteroposterior X-ray of the feet in 1977. (**E**) Frontal X-ray of the feet in 1977. (**F**) Lateral X-ray of the skull in 1978. (**G**) Frontal X-ray of the hands in 1979.

At the age of 9 years and 11 months, at a follow-up consultation, the following data were compiled from the physical examination: good nutritional state, stature 134 cm, weight 26 kg, presence of dysplastic facies, underdeveloped dentition, a systolic murmur identified at cardiac auscultation, pubic hair beginning to develop on the major labia and upper zone, observation of symmetric upper extremities with a proportionate shortening of the arm over the forearm during the examination of the musculoskeletal apparatus, prominence on the internal face of both elbows affecting pronation–supination movements, swelling of terminal phalanges of hands with watch-glass nails without local pain, and examination of lower extremities found bilateral genus valgus with widening of the distal feet and toenails of the same characteristics as those of the hands (Figure 2).

An X-ray of the skull showed dolichocephaly, elongated sella turcica and alterations of the sphenoid fissure. X-rays of the arms revealed bilateral dislocation of the radioulnar joint and acroosteolysis of the hands.

In 1977, the patient was diagnosed with idiopathic progressive acroosteolysis. The description of the case was performed by analyzing, in detail, the different clinical manifestations within their physiologic context and in a chronological order with the aim of evaluating the progressive nature of the syndrome.

## 3. Musculoskeletal Features

In 1978, the patient was referred to the traumatology service of the Nuestra Señora del Prado Hospital for the assessment of valgus flat foot that did not improve with orthopedic insoles. Finally, a surgical procedure was performed and the use of orthopedic footwear was recommended.

In 1987, due to the natural progression of the disease of her feet, the patient developed third degree pes cavus with irreducible claw toes and a marked equinus position of her left foot. She also presented with a persistence of recurvatum of the left knee and later developed the same problem on her right knee as well as bilateral genu valgum. Then, cephalic necrosis of the left hip appeared causing painful limitations with movements of the hip joint.

**Figure 3:**
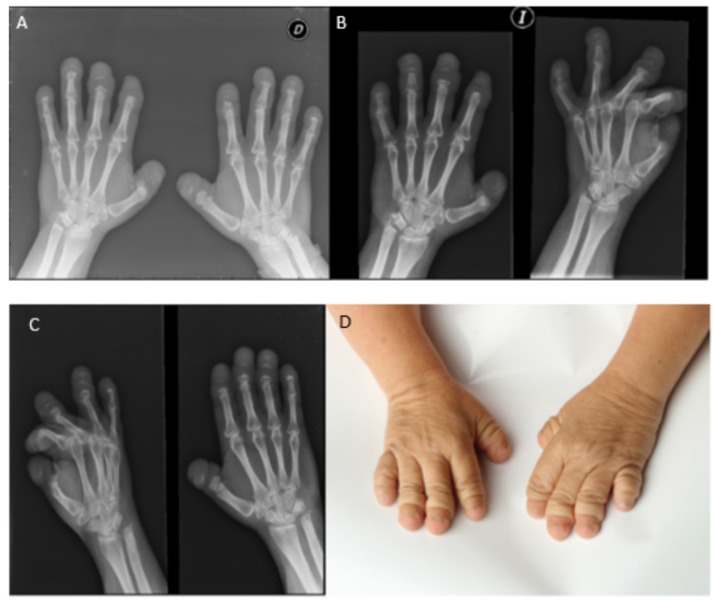

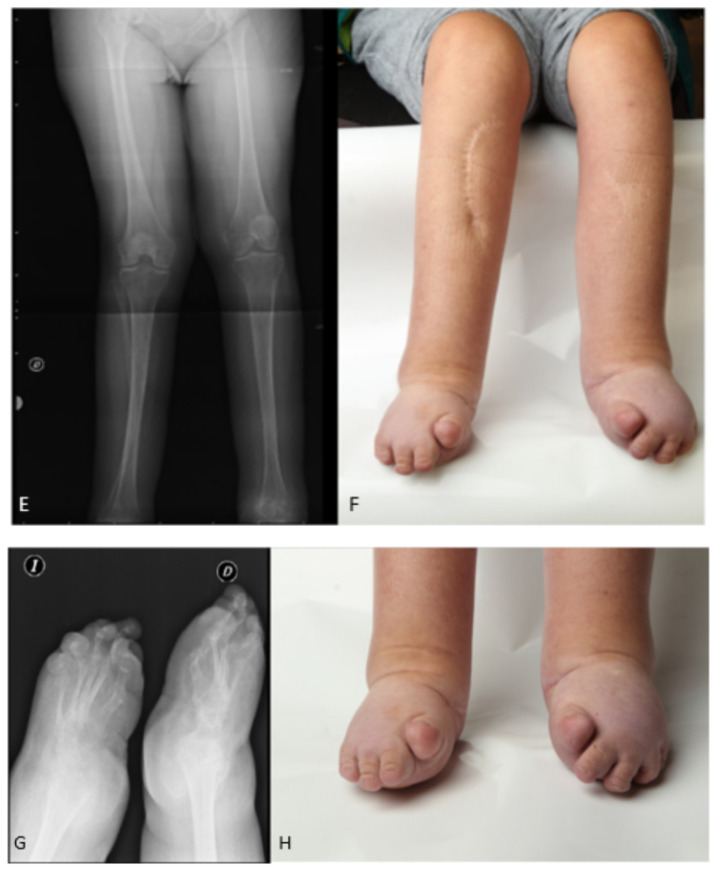

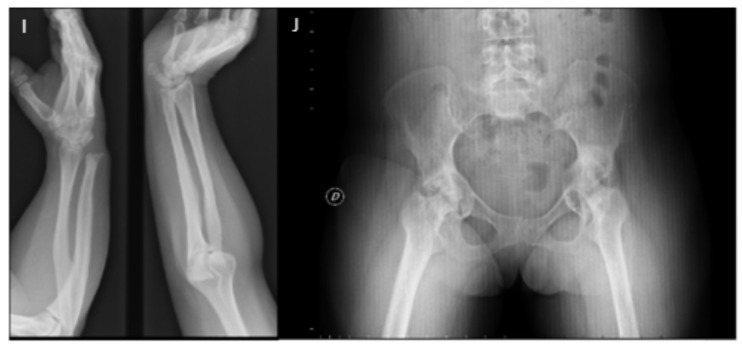
Radiologic controls. Phenotypic progression. (**A**) Radiological control hands in 2008. (**B**) Radiological control hands in 2011. (**C**) Radiological control hands in 2014. (**D**) Photography hands in 2021. (**E**) Serpentine fibula in 2002. (**F**) Photography legs in 2021. (**G**) Radiological control feet in 2014. (**H**) Photography feet in 2021. (**I**) Radiological control forearm in 2016. (**J**) Anteroposterior radiological control of the hip in 2013.

In 1993, the diagnosis worsened as the diseases progressed over time and deformities of the hands, feet, knees, hips and spine were present, accompanied by a considerable loss of strength (Figure 3).

In 1996, the patient visited the 12 de Octubre Hospital. During these visits, alterations of facial bones and the jaw were identified, and many teeth were found to be missing alongside alterations at the point of implantation. Other findings include early onset of hip arthritis, a complete disappearance of the third and second phalanges of the first and second fingers of her left hand, severe deformities of both feet that, alongside her knee deformities and a lumbar hyperlordosis, practically impeded walking. The patient walked with difficulty using crutches, although she began to use a wheelchair for longer distances.

In 1998, the patient underwent a bone biopsy, the results of which showed findings consistent with osteoporosis.

In 1999, new severe lesions of the distal phalanges of both feet were registered and a progressive destruction of areas of the tarsus, metatarsus and toes with spontaneous anquilosis of the left ankle in equinus position was found. The patient also had other congenital alterations such as a posterior dislocation of the radius of both elbows with functional limitations and a spondylosis of L5 that was identified during imaging controls. Later evaluations found a worsening of mobility and positioning of both feet, including the ankles, as well as a progressive destruction of both hips and spontaneous pain and contracture in flexion and internal rotation of the left hip. At this stage, both feet had lost all anatomical structure. Due to the auto aggressive nature of the syndrome, the patient was considered for surgical intervention for the insertion of hip prosthesis.

In 2008, the patient was diagnosed with bilateral collapse of femoral heads.

By 2012, the patient was limited to a wheelchair due to a complete inability to walk. There was also a progressive bone decalcification due to the lack of deambulation, further deformities in both hands, a left acetabular protrusion and bilateral coxarthrosis.

In 2013, a pelvis CT was obtained, which found a marked deepening of the femoral heads within the acetabular and bilateral protrusion; a diagnosis of Otto pelvis was made. The patient required evaluation for hip replacement. Her clinical condition was poor, with considerable disability requiring continuous assistance for everyday activities and transition to a motorized wheelchair to enable mobility. The patient complained of pain in the feet and edemas and of the inability to remain in a standing position due to the extensive destructuration of her coxofemoral joints.

In 2016, the clinical condition of her pelvis required referral to the Ramón y Cajal Hospital for evaluation of a possible bilateral hip replacement arthroplasty. Whilst the ongoing evaluation, the patient suffered a motor vehicle accident that has had a significant impact on her condition, notably worsening her general condition and her prospects of recovering any deambulation.

The accident suffered in 2017 marks a before and after in the progression of her disease. The patient was run over by a vehicle, taking a fall to the ground, which caused multiple lesions, mainly to her legs.

After the accident, the patient was referred to the pain management unit due to a severe worsening of the pain in her hips, ankle and left foot, although no fractures were identified.

By 2018, there was a slight improvement of the pain and the department of rehabilitation carried out a new evaluation of the possible impacts of a hip replacement but resolved to not move forward with the arthroplasty, based on the premise that the patient was unlikely to regain the ability to walk unassisted despite the surgical intervention.

In 2019, scleroderma was identified in association with the already present acroosteolysis.

Over the years of follow-up, bone density studies were carried out every 2–3 years by the department of rheumatology to study the progression of the disease regarding osteoporosis in order to adapt the established pharmacological treatment. Bone densitometry of the spine and the hip were performed. Table 1 and Table 2 show the data obtained in the latest studies.

In the densitometry control of 2012, there was an improvement in the parameters of bone density with regards to the 2005–2009 studies.

The last densitometry was performed in 2021. The results found that the bone density in the lumbar spine (L1–L4) was 0.673 gr/cm^2^ and 0.885 gr/cm^2^ in the left hip. Therefore, the diagnosis for the lumbar spine is compatible with osteoporosis and with osteopenia for the left hip.

## 4. Respiratory Features

In 2019, the patient was referred to the department of respiratory medicine for the study of dysphonia and a sensation of shortness of breath when speaking. It was decided to continue the study of her dyspnea in the sleep disorders’ unit to rule out nocturnal hypoventilation. A respiratory polysomnography study was carried out to establish baseline conditions.

Results showed a saturation tracing with several dips constituting a desaturation index of 15.1 per hour or registry, with a time of saturation below 90% (TC90) of 0.4%, and initial saturation of 95%, minimum saturation registered 80% and a mean saturation of 93.4%.

The mean heart rate was 83.2 bpm, with a maximum of 119 bpm and a minimum of 69 bpm. Regarding the respiratory signal, there was a tracing with apneas and hypopneas accompanied with oxygen desaturation that constituted an apnea–hypopnea index (AHI) of 15.9 events per hour of registry (changing from 5.1% of the registry in supine position, with a AHI of 19.6 and CT90.0%), with an index of central apneas of 0.3 per hour of registry, an index of obstructive apneas of 2.1 per hour or registry, an index of mixed apneas of 0 per hour of registry and an index of hypopneas of 13.5 per hour of registry. These results determine the existence of a mild to moderate sleep apnea–hypopnea syndrome without oximetric repercussions.

## 5. Ear, Nose and Throat Features

**Figure 4:**
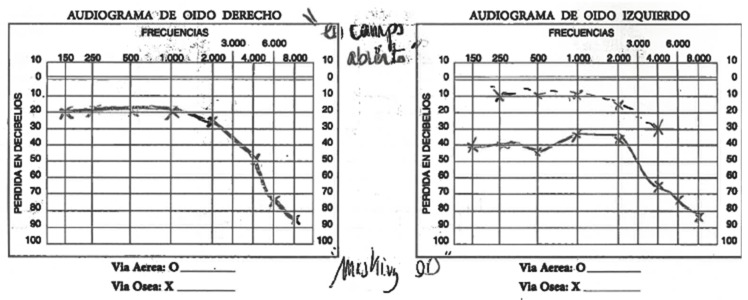
Audiogram of patient.

The patient has been followed-up with since 2019 for dysphonia and hypoacusia. During the physical exam, a small hyperemic polyp was identified on the anterior third of the right vocal cord. The patient was found to have a narrow nasal valve and a deep voice. An audiogram was performed, which detected signs of hypoacusia (Figure 4).

## 6. Gastroenterology Aspects

In 2011, an upper endoscopy identified signs of gastritis and a peptic-like lower esophageal ring.

In 2018, the patient was referred to the department of gastroenterology for the study of her dyspepsia. The patient complained of a longstanding intermittent diffuse abdominal discomfort and chronic constipation. Several lab and imaging studies did not identify any findings of interest.

## 7. Gynecology Aspects

The patient has been followed by the department of gynecology since 1994 for discomfort in both breasts.

In 1994, a bilateral mammogram report described very dense breasts with a predominant fibroglandular component compared to fatty tissue. Several cysts were seen in both breasts, the largest in the right breast of 18 × 14 mm. The patient was diagnosed as having bilateral fibrocystic mastopathy.

In 2001, a new bilateral mammogram detected several 2 cm nodules in the left breast, disperse bilateral calcifications that were more evident in the left breast in relation to sclerotic adenosis. A fine needle aspiration of the left breast allows for the pathology diagnosis of fibroadenoma.

In 2003, several cysts that resemble fibroadenomas were detected in both breasts.

In 2006, several new benign nodules were detected. Biopsies of both breasts were obtained, and the results confirmed the diagnosis of fibrocystic mastopathy.

In 2007, multiple bilateral cysts were observed, some with echogenic contents that gave them a solid aspect. A biopsy was taken for confirmation. The biopsy report stated duct ectasia and fibrocystic mastopathy.

In 2011, multiple simple cysts were found in both breasts, some of them with a less characteristic liquid content, that were most likely fibromas.

In 2016, a 2 cm well-defined solid nodule was found in the inferior quadrant of the left breast. The decision was made to carry out an ultrasound-guided core needle biopsy. The diagnosis was fibroadenoma.

## 8. Maxilofacial Features

**Figure 5:**
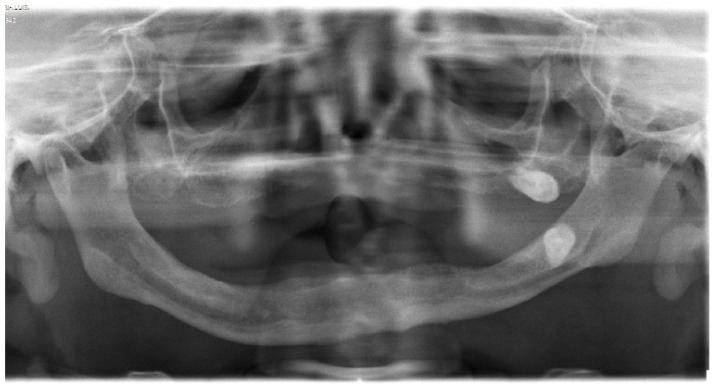
Orthopantomography of patient.

In 2011, the patient herself sought treatment as she had a complete loss of teeth and an extreme maxillary atrophy. She requested surgical intervention for the implant of teeth. However, an orthopantomography confirmed the high degree of atrophy, describing very fine alveolar edges and type IV maxillomandibular atrophy. Due to the state of the jaw bones, the surgical intervention was not considered (Figure 5).

## 9. Hematology Aspects

In 2018, during a routine lab analysis, a neutropenia of 900 was detected, leading to referral to the department of hematology. The patient did not have a history of fever or recurrent infections.

She was diagnosed with a mild–moderate intermittent cyclic neutropenia of an unknown etiology, although this was possibly idiopathic, autoimmune or drug-induced.

## 10. Psychiatric Aspects

After the accident that took place in 2017, the patient required evaluation by the psychiatric team. She suffered from anxiety that negatively impacted her daily life. Her extreme dependency on other people for all activities made her feel sad and the appearance of nightmares about the accident caused her sleep disturbances. She described feelings of apathy regarding the impossibility of recovering her former life. The patient required medication in order to sleep and expressed difficulties for paying attention and concentrating and a certain hopelessness about her current situation. She presented no sensory perceptual alterations, and her judgement of reality was preserved.

The patient was diagnosed with a prolonged adjustment disorder with clinical manifestations of depression and anxiety secondary to the pain and loss of autonomy after her traffic accident.

## 11. Psychosocial Aspects and Lifestyle

The patient experienced significant changes in her lifestyle and the psychosocial spheres of her life after the 2007 accident. The functional limitations of her lower extremities did not allow her to carry out everyday activities independently or with any degree of autonomy. Activities such as bathing, cooking, getting dressed, and driving were activities she once carried out daily but were no longer possible. She could not drive and wore nighttime diapers to avoid unnecessary painful bathroom visits. She used a transportation crane for most of her mobilization. The levels of depression and anxiety that arose from these situations were high.

To evaluate the degree of depression, we used the Patient Health Questionnaire (PHQ-9) [23], obtaining a score of 18/29, which indicated a moderate to severe level of depression requiring pharmacological and interventional treatment. The level of anxiety was assessed using the General Anxiety Disorder scale (GAD-7) [24], where she scored 21/21, indicating severe symptoms of anxiety. We also used the Health Questionnaire SF-36 [25] to assess her quality of life, obtaining scores of 24 in the section of physical health and 25 in the section of mental health. Both scores below 30 confirmed a subpar quality of life.

## 12. Milestones

Table 3 shows the main milestones in the description of the case.

## 13. Diagnosis Related Problems, Differential Diagnosis and Prognosis

When it comes to rare diseases, one of the main problems that often arises is the late diagnosis. Approximately 80% of rare diseases have a genetic origin, thus, most of the time, the definitive diagnosis is only obtained after genetic sequencing. A prior diagnostic suspicion and a careful consideration of the observed clinical findings are essential. In the case of this specific disease, there is a useful clinical tool that was designed by Brennam et al. [9] that established inclusion criteria based on physiological parameters and genetic inheritance. The complexities in the diagnosis of Hajdu–Cheney syndrome arise when there are clinical findings that are also present in other diseases, leading to several crossover points that increase the possibilities and the need for a differential diagnosis. Specifically, in the case we have presented, the first diagnostic suspicion that arises is pycnodysostosis.

Pycnodysostosis [26] is also a rare disease that is caused by mutations in the gene that codes cathepsin K (located on 1q21). The most frequent clinical manifestations include osteosclerosis, short stature, acroosteolysis of the distal phalanges, bone fragility, dysplasia of the clavicula, cranial malformations such as a large skull, wormian bones and persistence of the anterior fontanelle and a small jaw. Dental anomalies as well as irregular and brittle nails are often seen. The disease is not progressive.

As one can see, there are clinical links between the two syndromes, which complicates making the diagnosis. The study of the complete phenotype of the disease, the observation of the clinical findings and, finally, genetic sequencing, are the key elements needed to arrive at the definitive diagnosis.

The prognosis of patients affected by HCS depends on the severity of the disease, clinical complications and the degenerative progression of each patient. The generalized osteoporosis and the development of acroosteolysis will likely cause fractures, limit deambulation and lead to dependency for basic everyday activities. The most frequent complications in this disease include basilar invagination, which causes neurological alterations, or chest wall deformities that give way to ventilation restriction.

## 14. Therapeutic Interventions

Table 4 shows the therapeutic interventions to which the patient has been subjected.

## 15. Discussion

The case we have described presents the three main features of Hajdu–Cheney syndrome.

The phenotypic variability [8,27,28], a consequence of the variability of expression of NOTCH2, is evident when comparing this case to other cases diagnosed with HCS that have been published in the scientific literature. Cases such as those presented by Swan et al. [29], Ades et al. [12] and Takatani et al. [30] all show differences in their physical appearance and clinical presentation despite having the same diagnosis.

The degenerative nature [31] is evident in most of the radiological findings that we analyzed and in the continuous deterioration of the patient’s autonomy. This fact was also described by Harnasch [32] in the description of their case, which reports that the patient began to suffer a gradual loss of strength until becoming completely dependent for everyday activities.

Similar cases of the acroosteolysis of the distal phalanges and the generalized osteoporosis that this patient presented are described in the majority of cases diagnosed with HCS that are published to date. The cases reported by Rosenmann et al. [33], Elias et al. [34] and Bruckner et al. [35] suggest that the most prevalent signs of HCS are acroosteolysis and generalized osteoporosis.

Diagnostic reasoning is guided by observation and the radiological findings [21]. Brennam et al. [9] created a clinical tool to facilitate the diagnosis of this syndrome. The tool includes a list of physiological parameters and genetic inheritance as inclusion criteria for HCS. Among the included features are acroosteolysis, premature loss of teeth, short stature and dysplastic facies. The case we present in this paper presents all the above mentioned features, and therefore, complies with the inclusion criteria and positively guides the case towards the diagnosis of HCS.

During the first medical visits, the suspected possible diagnosis was pycnodysostosis [26] due to the phenotypic similarities that were found. A similar debate occurred in the case reported by Herrmann et al. [36].

Once a suspected diagnosis is established, the definitive diagnostic confirmation must be determined by Gibofsky genetic sequencing [20]. In this case, a constitutional cytogenetic study in peripheral blood was performed in 1998. The results after the analysis of 15 metaphases showed a karyotype without numeric or structural anomalies. In 2020, considering the lack of further genetic studies, the patient was referred to the genetics department for complete genetic sequencing.

Hajdu–Cheney syndrome generally follows an autosomal-dominant inheritance pattern, as reported by Majewski et al. [3]; however, sporadic cases do exist, such as the case presented by Descartes et al. [4].

Over the course of the follow-up of this patient, a series of findings comparable to previously described cases of this disease have reinforced the available knowledge on this disorder. At the same time, new aspects arise that may serve as a guide for future lines of research.

The musculoskeletal alterations that are presented in this case, such as the generalized osteoporosis and the acroosteolysis of the distal phalanges, are two of the main manifestations of this syndrome that are also present in the majority of cases that have been studied, as Letchumanan et al. [15], Stathopoulos et al. [16] and Nunziata et al. [37] report in their publications.

Siklar et al. [38] described the link between growth hormone and short stature in patients with HCS, a noticeable aspect in this case. Characteristics of a dysplastic facies at a cranial level, including dolichocephaly, elongated sella turcica and alterations of the sphenoidal fissure, are present in cases such as the case Hajdu et al. [5] described in their first description of the syndrome, in a patient with cranial alterations.

The bilateral genu valgum that our patient presented has previously been described in different occasions by Willians [39] and Weleber et al. [40].

A frequent finding in Hajdu–Cheney syndrome is watch-glass nails, an element which is present in this case and that has also been described in the literature by Rosenmann et al. [33], among others. They present a patient with a particular finding that is also seen in this case: the implication of the radial head.

The disease progresses over time, a fact which is most noticeable in the deterioration of the musculoskeletal apparatus. This patient presented deformities of the hands, such as those described by Jiménez et al. [41] Shurtleff et al. [42] Brown et al. [10] and Ventosa et al. [43]; of the feet, such as those described by Greenberg et al. [44] and Colmenares Roldán et al. [45]; of the knees, as discussed by Weleber et al. [40]; and of the spine, reported by Vissarionow et al. [46] and Chawla [47]. This worsening at a skeletal level is accompanied by a considerable loss of strength that increases the disabling nature of the syndrome.

The presence of hyperlordosis and hyperkyphosis in this case made walking progressively difficult. Initially, the patient was able to manage with the help of crutches, but this became no longer possible and she progressed to requiring a wheelchair for any kind of mobilization. In the case described by Rosenmann et al. [33], the same clinical findings were present.

The disabling coxarthrosis with acetabular protrusion is another sign of the degenerative nature of the disease as it can be seen how it is worsening as time passes. Otto pelvis is a sign detected in this case that has not been previously reported in any of the studied cases to date.

Reviewing the complete radiological study that is available in the patient’s clinical record, there is a finding that is worth noting, although it was not referenced in any of the clinical reports that we reviewed: the presence of serpentine fibula. This finding gives the case certain peculiarities that should be mentioned.

For some time, patients who presented serpentine fibula were diagnosed with serpentine fibula and polycystic kidney syndrome. Considering their phenotypes and the evident differences between those patients and patients diagnosed with HCS, the two syndromes we considered were completely independent diseases. Currently, owing to the advances in genetics, it has been demonstrated that the gene responsible for Hajdu–Cheney syndrome and the gene responsible for serpentine fibula and polycystic kidneys syndrome are one and the same. Therefore, the two entities constitute a single disease. Studies such as those by Fryns et al. [48], Ramos et al. [49], Currarino et al. [18], Isidor et al. [19] and Gray et al. [50] confirm that both syndromes are actually the same disease and that the presence of serpentine fibula is just another manifestation of HCS. Despite this fact, it is of interest to study the specific cases that present the association of serpentine fibula and polycystic kidneys. The patient described in this paper has not yet had the necessary studies to determine the possible existence of cysts in her kidneys.

Biopsies are a frequent method used for the analysis of bone alterations in this syndrome. Elias et al. [34] and Iwaya et al. [51] analyzed bone samples of their patients and describe findings similar to those of this case. Vascular and perivascular proliferation with a scarcity of bone trabeculae compatible with osteoporosis (the diagnosis of this patient) are related to the evidence presented by Brown et al. [10] in their study where they carry out a complete description of the syndrome, arguing that these patients present with low bone density and a deficit in bone formation.

In their study, Sargin et al. [17] mention the congenital heart alterations that can be found in HCS. During the physical examination of our patient at nine years of age, doctors identify a systolic heart murmur on auscultation, the same finding as in the case reported by Herrmann et al. [36].

The sensation of dysphonia and shortness of breath when talking leads to the diagnosis of a mild to moderate sleep apnea–hypopnea syndrome without oximetric repercussions. In the literature, there are cases that present with dyspnea as a consequence of chest wall deformities that limits ventilation capacity and increases the likelihood of infections. The cases described by Williams [39] and Sasaki [52] present these elements of dyspnea and infections.

Hearing loss is another frequent sign of this syndrome, and it is present in this case and also in cases as described by Herrman et al. [36]).

At a gastrointestinal level, intestinal malrotation and dysphagia are elements that are present in cases such as those described by Williams [39] and N. Hajdu [5] that belong to the wide spectrum of signs and symptoms of this syndrome. The digestive findings identified in this case are chronic constipation and gastritis.

Fibrocystic mastopathy and fibroadenomas have not been reported in any of the cases in the literature to date. One of the few descriptions of gynecological issues in this syndrome was carried out by Nozaki [53], who presented the case of a woman with premature ovarian insufficiency.

The premature loss of teeth is another typical sign of HCS that has been considered on several occasions by different authors, including Shaw [54] and Lee et al. [55]. Bazopoulou-Kyrkanidou et al. [14] and Antoniades et al. [56] also have studies on dental alterations that are worth noting. Another significant paper regarding dental restoration in patients with this syndrome was published by Vingerhoedt et al. [57].

The case of neutropenia without fever or the presence of infections that were found in this patient have not been previously described in relation to HCS; however, the cases reported by Shurtleff et al. [42] and Chawla [47] present recurrent infections.

In this case, there is a clear before and after in the progression of the disease related to the traffic accident the patient suffered in 2017. The patient was run over by a vehicle, causing her to fall and suffer multiple lesions. This event had a noticeable negative effect on her day to day life, leaving her completely disabled for most daily activities, increasing her level of dependency and further developing mental disorders such as stress, anxiety and depression.

At this point, it is important to note that there are studies that justify the psychological burden and the increase in mental disorders that occur in patients with rare diseases [58].

With regard to treatment, several aspects can be analyzed: pharmacological, surgical and self-care.

The studies on pharmacological treatments for this syndrome do not present clear evidence on their efficacy. Relevant studies on pharmacological treatments for HCS include that by Sakka et al. [59], who worked on a therapy that uses bisphosphonates and found oscillations in bone mineral density indexes of the lumbar spine. At the beginning of the study, the values decreased, then increased in response to treatment, but the effect did not persist after its interruption. Pittaway et al. [22] also studied bisphosphonates but obtained different results for each patient that were dependent on age. Adami et al. [60] tried obtaining an increase in bone mineral density with denosumab but acroosteolysis persisted. Tsinopoulou et al. [61] and Al-Mayouf et al. [62] experimented with pamidro-nate, without achieving a curative result. Hwang et al. [63] tried to slow down the process of bone degradation using zoledronic acid, and in 2007 and 2008, McKiernan et al. [64,65] carried out a pharmacological study to treat osteoporosis in HCS with an antiresorption and anabolic therapy. Treatment with bisphosphonates has a negative impact on the development of surgical interventions at the oral level because they hinder healing and increase the risk of osteonecrosis of the jaw [66].

The surgical intervention of flat feet that was performed in 1978 is part of the different surgeries that are mentioned in this case. Studies on surgical interventions in HCS are those by Mattei et al. [67] on cervical kyphotic deformities in patients with osteoporosis and by Murtagh-Schaffer et al. [68] on spinal reconstruction. In this case, bilateral arthroplasty hip replacement was a treatment option that was considered but that was finally not pursued for clinical reasons. The papers by Yamaguchi et al. [69] and August et al. [70] on the specific indications for the treatment of patients with HCS before surgery must also be mentioned.

Current treatment for HCS is mainly centered on the management of the complications that arise and on the treatment of the underlying problems in order to improve the patient’s quality of life and life expectancy. In this case, several self-care recommendations were made, including maintaining an active as possible lifestyle, both physically and intellectually, and avoiding overweight as a preventative method against disease progression.

## 16. Limitations

The limitations we have identified in the development of this case study are directly related to the low specificity of the clinical studies that were performed. The lack of a general perspective on the progressive nature of the disease and a lack of awareness with regard to the disease itself have a negative impact on the follow-up of the case.

It would be of scientific interest to perform a genetic study in order to identify the exact underlying mutation and relate it to the phenotype. Confirming whether or not the patient has polycystic kidneys would provide vital information for follow-up. Regarding diagnostic testing, an updated complete radiological control to assess the possibility of basilar invagination, a typical complication in this syndrome, is recommended. Another frequent complication is ventilation restriction due to thoracic deformities, so this should also be considered. At present, the patient complains of a severe pain in the occipital area that does not resolve with her usual treatment. She also states that she experiences excessive tiredness with minimal efforts. Both signs are compatible with the most frequent complications of this syndrome, as have been previously mentioned.

## 17. Conclusions

The study of this case, in the context of general knowledge on Hajdu–Cheney syndrome, reinforces and justifies its three main features: phenotypic variability, age-dependent progression and acroosteolysis of the distal phalanges and generalized osteoporosis, all elements considered obligatory inclusion criteria for the diagnosis of this disease.

However, a complete and updated description of the phenotype of this syndrome, which includes a large sample of cases, is still needed.

The study of the hematological effects of HCS may constitute a possible future line of research of this syndrome.

## Figures and Tables

**Table 1:** Evolution of the reviewed parameters of bone densitometries of the spine.

Date	Age	Dmo (g/cm^2^)	Difference (%)	Difference/DE
10 October 2005	38	0.819		
7 May 2008	40.5	0.872	6.5	5.3
2 December 2011	44.1	0.914	11.6	9.5
29 May 2014	46.6	0.856	4.5	3.7
10 May 2016	48.5	0.814	−0.6	−0.5

**Table 2:** Evolution of the reviewed parametres of bone densitometries of the hip.

Date	Age	Dmo (g/cm^2^)	Difference (%)	Difference/DE
10 October 2005	38	0.846		
2 December 2011	44.1	1.933	128.5	77.6
29 May 2014	46.6	1.508	78.3	47.3
10 May 2016	48.5	1.300	53.7	32.4
10 December 2018	51.1	1.035	22.3	13.5

**Table 3:** Main milestones in the description of the case.

Important Milestones	Year
Birth	1967
First medical registry	1973
Diagnostic suspicion	1973
Definitive diagnosis	1977
Pes valgus surgery	1978
Walks using crutches	1996
Diagnosis of breast fibroadenomas	2001
Begins treatment with bisphosphonates	2006
Wheelchair	2012
Ends treatment with bisphosphonates	2012
Evaluation for possible hip replacement	2016
Traffic accident	2017
Dependency for activities of daily living	2017
Diagnosis of prolonged adjustment disorder with clinical manifestations of depression and anxiety secondary to the pain and loss of autonomy after accident	2017
Diagnosis of neutropenia	2018
Diagnosis of OSAHS	2019
Scleroderma related to HCS	2019

**Table 4:** Therapeutic interventions.

PHARMACOLOGICAL	
PAINKILLERS	
DICLOFENAC 50 mg every 12 h	Treatment for pain management is established following the recommendations of the World Health Organization (WHO) in their analgesic ladder. The strategy consists of a series of drugs administered regularly and rescue medications for the occasions when routine treatment is insufficient for pain control.
PARACETAMOL 650 mg every 4 h	
TRAMADOL HYDROCHLORIDE 50 mg every 6 h (rescue medication)	
DAFALGAN (paracetamol) 1 gr every 6 h (rescue medication)	
METAMIZOLE 575 mg every 6 h	
FENTANYL patch 12 mcg every 72 h	
TRAMADOL HYDROCHLORIDE 200 mg at breakfast	
CELECOXIB 200 mg every 24 h	
SUPPLEMENTS	
OSSOPAN^®^ 400 mg every 24 h	Supplements of calcium and vitamin D are administered together to boost their effect against osteoporosis.
SUPRADYN^®^ PROTOVIT 9 drops every 24 h	
ORAL CALCIUM 1 gr every 24 h	
VITAMIN D3 8000 U every 24 h	
IDEOS^®^ 500 mg every 12 h	
HIDROFEROL^®^ 0.266 mg every 15 days	
ANTI-ACIDS	
OMEPRAZOLE 20 mg every 24 h	Due to the diagnosis of gastritis and the polypharmacy the patient receives, an antiacid is required to protect her stomach lining.
BISPHOSPHONATES	
ACREL^®^ 35 mg weekly (risedronic acid)	The treatment with bisphosphonates began in 2006 and was cut off in 2017 after an improvement in densitometric parameters.
ADROVANCE 70 mg weekly (alendronic acid/colecalciferol)	
ANTIDEPRESSANTS	
XERISTAR^®^ 60 mg every 12 h (duloxetine)	Antidepressant treatment begins in 2017 after the diagnosis of a prolonged adjustment disorder with clinical manifestations of depression and anxiety secondary to the pain and loss of autonomy after her traffic accident.
DEPRAX^®^ 100 mg every 24 h in the evening (sertraline)	
DIAZEPAM 5 mg every 12 h	
STILNOX^®^ 10 mg every 24 h in the evening (zolpidem tartrate)	
VENLAFAXINE 37.5 mg every 24 h	
ZARELIS^®^ 75 every 24 h	
LAXATIVE	
MOVENTING^®^ 25 mg at breakfast (Naloxegol)	The treatment of chronic constipation requires the regular use of laxatives.
SURGICAL	
Pes planus valgus	Surgical intervention in 1978 of the patient’s pes planus valgus determines the use of orthopedic insoles and footwear.
Surgical lumpectomy of a benign breast lesion	In the context of a fibrocystic mastopathy with fibroadenomas, surgical intervention is required for the correct evaluation of a breast lump.
SELF-CARE	
Daily basic walk Avoid overweight Maintain physical activity Intellectual activity	Self-care recommendations are designed to maintain as much physical and intellectual activity as possible and to avoid overweight as a preventative method against disease progression.

## Data Availability

Not applicable.

## Abbreviations

HCS	Hajdu-Cheney syndrome
CT	Computed tomography
VAS	Visual Analog Scale
AHI	Apnea–hypopnea index
BPM	Beats per minute
FEV	Forced expiratory volume
FVC	Forced vital capacity
IT	Inspiration time
PHQ-9	Patient Health Questionnaire
GAD-7	General Anxiety Disorder scale
OSAHS	Oximetric sleep apnea–hypopnea syndrome
